# Sex Differences in the Response to Viral Infections: TLR8 and TLR9 Ligand Stimulation Induce Higher IL10 Production in Males

**DOI:** 10.1371/journal.pone.0039853

**Published:** 2012-06-29

**Authors:** Maria Gabriella Torcia, Lucia Nencioni, Ann Maria Clemente, Livia Civitelli, Ignacio Celestino, Dolores Limongi, Giulia Fadigati, Eloisa Perissi, Federico Cozzolino, Enrico Garaci, Anna Teresa Palamara

**Affiliations:** 1 Department of Clinical Physiopathology, University of Firenze, Firenze, Italy; 2 Department of Public Health and Infectious Diseases, “Sapienza” University of Rome, Rome, Italy; 3 Department of Experimental Medicine and Biochemical Sciences, University of Rome “Tor Vergata,” Rome, Italy; 4 Department of Public Health and Infectious Diseases, Institute Pasteur Cenci Bolognetti Foundation, “Sapienza” University of Rome, Rome, Italy; 5 San Raffaele Pisana Scientific Institute for Research, Hospitalization, and Health Care, Rome, Italy; Johns Hopkins University - Bloomberg School of Public Health, United States of America

## Abstract

**Background:**

Susceptibility to viral infections as well as their severity are higher in men than in women. Heightened antiviral responses typical of women are effective for rapid virus clearance, but if excessively high or prolonged, can result in chronic/inflammatory pathologies. We investigated whether this variability could be in part attributable to differences in the response to the Toll-Like Receptors (TLR) more involved in the virus recognition.

**Methods:**

Cytokine production by peripheral blood mononuclear cells (PBMCs) from male and female healthy donors after stimulation with Toll-like receptors (TLR) 3, 7, 8, 9 ligands or with viruses (influenza and Herpes-simplex-1) was evaluated.

**Results:**

Compared to females, PBMCs from males produced not only lower amounts of IFN-α in response to TLR7 ligands but also higher amounts of the immunosuppressive cytokine IL10 after stimulation with TLR8 and TLR9 ligands or viruses. IL10 production after TLR9 ligands or HSV-1 stimulation was significantly related with plasma levels of sex hormones in both groups, whereas no correlation was found in cytokines produced following TLR7 and TLR8 stimulation.

**Conclusions:**

Given the role of an early production of IL10 by cells of innate immunity in modulating innate and adaptive immune response to viruses, we suggest that sex-related difference in its production following viral nucleic acid stimulation of TLRs may be involved in the sex-related variability in response to viral infections.

## Introduction

Toll-like receptors (TLRs) are germline-encoded pattern recognition receptors (PRRs) that play a central role in host cell recognition and responses to microbial pathogens. They recognize conserved molecules associated with microbial pathogens, known as microbe-associated molecular pathways (MAMPs). A number of TLR family members have evolved to recognize various forms of viral nucleic acid in endosomic or lysosomal compartment of infected cells: TLR3 senses dsRNA produced during replication of RNA and DNA viruses. TLR7 and TLR8 recognize ssRNA, which is rich in uridine or uridine/guanosine. TLR9 recognizes the genome of DNA viruses containing unmethylated CpG DNA motifs [1]. Upon MAMP recognition, TLRs induce the activation of intracellular signaling cascades that result in the production of both pro- and anti-inflammatory cytokines and chemokines. A common theme for all nucleic acid sensing TLRs is the induction of type I IFNs, which act at multiple stages of the immune response and induce the expression of hundreds of genes involved in antiviral resistance [2]. However, the major TLRs contribution to the development of antiviral immune response is due to their control of dendritic cells (DCs) function [1]–[3]. TLR-dependent DCs maturation appears to be critical for the priming of both CD4 and CD8 T cells during many viral infections. For example, TLRs can upregulate the production of IL12, IL23 and IL27 to induce the differentiation and maintenance of T and B cells promoting specific immune responses [3].

During infection, induction of inhibitory immune pathways mainly mediated by IL10 production by DCs can also be observed [4]–[6]. The early production of IL10 may strongly affect both innate and adaptive immune response by inhibiting the expression of co-stimulatory molecules, limiting the production of inflammatory cytokines and chemokines, and by inducing the activation of regulatory T cells (Tregs) that inhibit effector T cell activity [7]. Several viruses have been reported to induce elevated levels of IL10 production by the cells of innate and adaptive immunity, which correlate with the impairment of T-cell responses, resulting in the decreased ability to control viral replication [8]–[11].

It is well known that manifestations of viral infections differ between women and men, and marked sex differences have been described in the incidence and course of different RNA or DNA virus infections [12]–[17]. Many studies have proposed that the greater humoral and cell-mediated immune responses of females to viral antigens play an important role in determining the gender variability in viral infections [12], [18] and, in females, such “immunological power” creates a double-edge sword, being beneficial against infectious diseases, but detrimental in terms of increased development of autoimmune diseases [19].Although essential in shaping the amplitude and quality of specific immune response, sex-related differences in the mechanisms of innate immunity and in particular in TLR pathways have been poorly explored [20], [21]. In this paper we investigated the production of pro-inflammatory and anti-inflammatory cytokines in groups of healthy males and females in response to stimulation of the TLRs more involved in viral recognition and found major differences in the production of IL10, a cytokine serving as a negative regulator of the response of both innate and adaptive immune cells, with males always being higher producers than females.

## Results

### Sex Related Differences in IFN-α Production in Response to TLR Ligands

PBMCs, isolated from healthy males (n = 20, mean age 42±13) and females (n = 40, mean age 47±14), were stimulated with Imiquimod as TLR7 ligand, ssRNA40 as TLR8 ligand, Poly (I:C) as TLR3 stimuli or CpG-ODN 2006 as TLR9 stimuli. Culture supernatants were collected after 48 hours and stored at −20°C.

**Figure 1:**
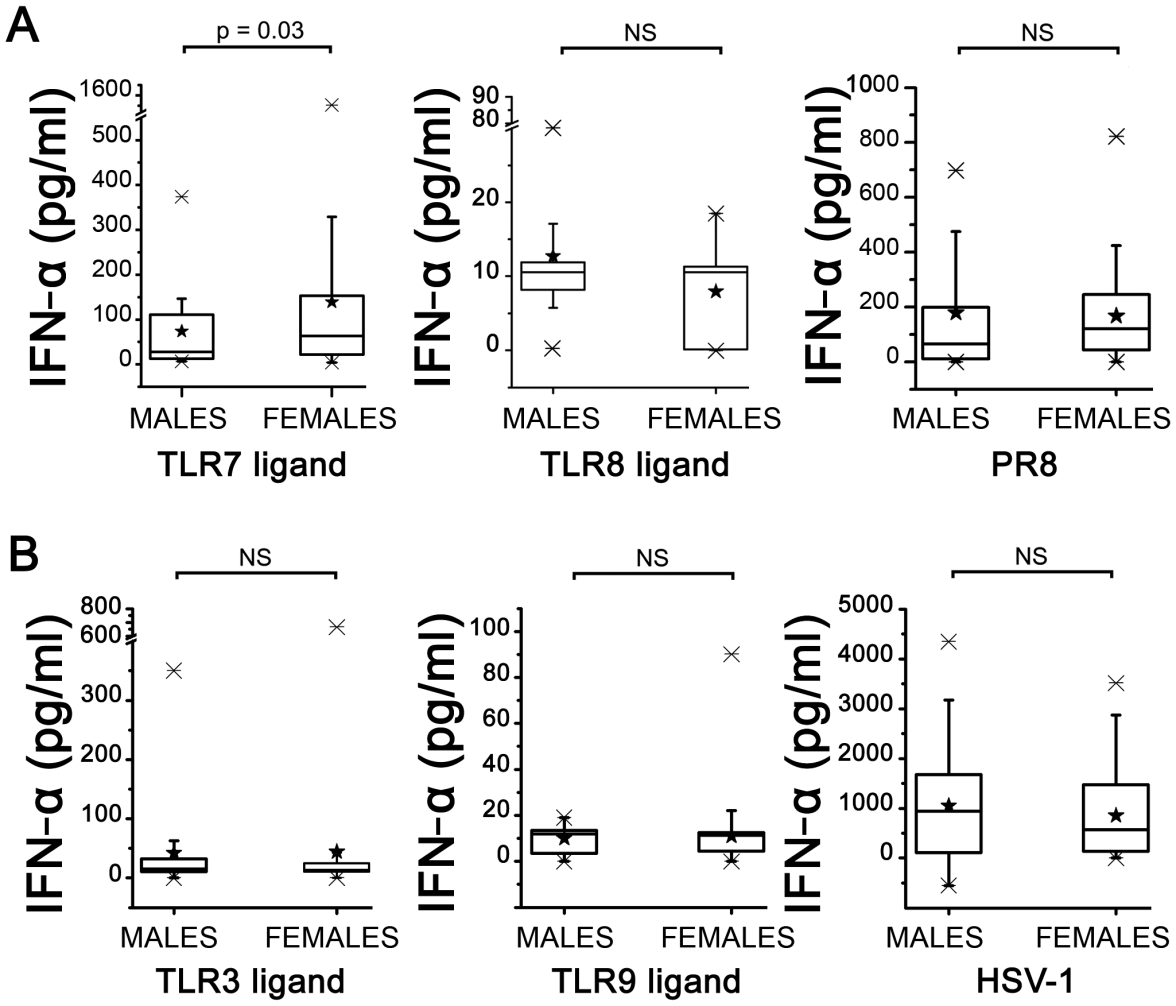
IFN-α production by PBMCs following TLR stimulation or infection with influenza virus or HSV-1. Panel A: PBMCs from males (n = 20) or from females (n = 40) were stimulated with Imiquimod as TLR7 ligand, ssRNA40 as TLR8 ligand or infected with influenza virus (PR8). Panel B: PBMC from males or females were stimulated with Poly(I:C) as TLR3 ligand, ODN 2006 as TLR9 ligand or infected with HSV-1. IFN-α was measured in culture supernatants by immunoplex array. Data for each donor are calculated as mean IFN-α production in stimulated cultures – mean IFN-α production of unstimulated cultures. Data are presented as box-and-whisker plots, with boxes extend from the 25^th^ percentile to the 75^th^ percentile, with a horizontal line at the median while whiskers extend to the lowest and highest data point. Black stars indicate mean values. Statistical analysis was performed by two-tailed non parametric Mann-Whitney test. Significance levels were fixed at p<0.05. IFN-α concentrations in unstimulated or mock-infected cultures ranged between 2 and 30 pg/ml (mean 9.2±1.4). No significant differences between the groups were detected.

Analysis of data of IFN-α concentrations in culture supernatants revealed significant differences between females and males after stimulation with Imiquimod: the mean production of IFN-α was almost the double in the female group (Figure 1, panel A) compared to males. In contrast, by using ssRNA40 as TLR8 ligand we did not detect differences in IFN-α production between males and females (Figure 1, panel A) suggesting that, despite their phylogenetic relationship, the sensitivity of the two viral RNA sensors could be differently modulated through sex-related pathway. Next we studied whether the reported differences between males and females in IFN-α production following TLR7 stimulation could be correlated with “in vivo stimulation” by sex hormones and/or Cortisol. The data obtained did not show any correlation with plasma levels of DHS or TES levels in the male group (Spearman K = 0.179, p = 0.46) or correlation with E2 plasma levels in the female group (Spearman K = −0.05 p = 0.8). No correlation was found with plasma levels of COR in the two groups (data not shown). These data suggest that, according to other studies [22], plasma levels of sex hormones do not influence the sensitivity of TLR7 to Imiquimod.

To investigate the biological relevance of the data obtained using TLR7 and TLR8 ligands, we infected PBMCs of the same subjects with influenza virus (PR8), a RNA virus known to induce IFN-α production through interaction with TLR7/8 [23], and measured the production of IFN-α 24 hours post infection (p.i). Figure 1, panel A shows that the production of IFN-α following influenza virus infection did not differ significantly between males and females suggesting that, in addition to TLR7/8, influenza virus induces IFN-α production through pathways not influenced by sex-related factors.

We also studied IFN-α production following infection of PBMCs with HSV-1, a virus largely spread among the adult population, as representative of DNA virus and compared IFN-α production with those obtained by triggering two TLRs reported as crucial for immunity to HSV-1, TLR9 and TLR3 [24]–[26]. IFN-α was measured in culture supernatants of PBMCs from males and females after 48 hours of infection with HSV-1 or stimulation with Poly (I:C) as TLR3 ligand and CpG-ODN 2006 as TLR9 ligand. Figure 1, panel B shows that IFN-α production did not differ significantly in the two groups following TLR3, TLR9 stimulation or following HSV-1 infection.

On the whole these data suggest that the increased production of IFN-α following TLR stimulation does not represent the single mechanism on the basis of the lower susceptibility of females to viral infection.

### Production of IL12p40, IL12p70, IL13, TNF-α in Response to TLR Ligands or Viral Infections

Then the levels of cytokines directing the type of immune response, IL12p40, IL12p70, IL13, and the pro-inflammatory cytokyne TNF-α were measured in all the supernatants recovered from PBMCs stimulated with TLR3, TLR7, TLR8, TLR9 ligands or from PBMCs infected with influenza virus or HSV-1. With the exception of a higher production of TNF-α following stimulation with TLR3 ligands in the male group compared to females (Figure S1), no statistically significant differences were found between males and females.

### IL10 Production in Response to Ligands of TLRs Sensing Viral Nucleic Acids and to Virus Infection

It is known that several viral infections induce inhibitory pathways, mainly mediated by IL10 production by immune cells as part of their strategies to escape immune surveillance. An early production of IL10 by monocytes and DCs in fact may strongly affect both innate and adaptive immune response by limiting the maturation of DCs and by inducing the activation of Tregs that inhibit effector T cell activity [7]. We thus analyzed whether the stimulation of the TLRs more involved in viral recognition induced the production of IL10 and whether males and females produced this inhibitory cytokine differently.

**Figure 2:**
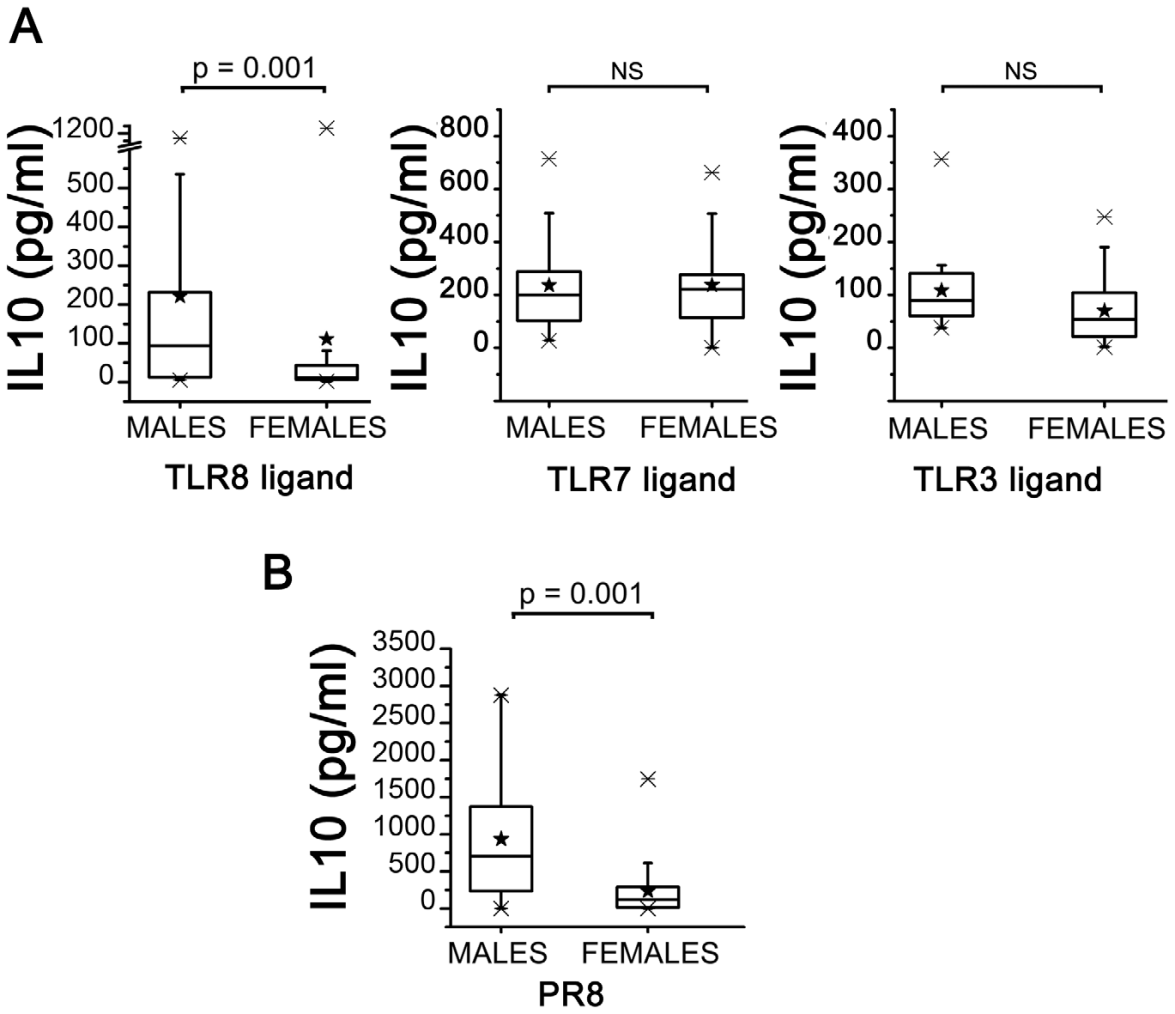
IL10 production by PBMCs following TLR8, TLR7, TLR3 stimulation or infection with influenza virus. PBMCs from males (n = 20) or from females (n = 40) were stimulated with ssRNA40 as TLR8 ligand, Imiquimod as TLR7 ligand, Poly(I:C) as TLR3 ligand (Panel A) or infected with PR8 influenza virus (Panel B). IL10 was measured in culture supernatants by immunoplex array. Data for each donor are calculated as mean IL10 production from stimulated cultures – mean IL10 production of unstimulated cultures. Data are presented as box-and-whisker plots, with boxes extend from the 25^th^ percentile to the 75^th^ percentile, with a horizontal line at the median while whiskers extend to the lowest and highest data point. Black stars indicate mean values. IL10 concentrations in unstimulated or Mock-infected cultures ranged between 2 and 68 pg/ml (mean 28.3±1.6). Statistical analysis was performed by two-tailed non parametric Mann-Whitney test. Significance levels were fixed at p<0.05.

Figure 2, panel A shows the results obtained by stimulating PBMCs with ligands of TLRs sensing viral RNA (TLR3,7,8): the stimulation of TLR8 with ssRNA40 induced a significant higher production of IL10 in the male compared with female group. In contrast, no differences in IL10 production were detected following stimulation of PBMCs from males or females with Imiquimod as TLR7 ligand. Once more, despite the close phylogenetic relationship between TLR7 and TLR8, these data suggest that different biochemical pathways, both influenced by sex, could be activated following their stimulation. The stimulation of TLR3 (which senses the dsRNA intermediate product) with Poly (I:C) also induced an excess production of IL10 in the male group compared to females but the results did not reach the significance.

To check the biological relevance of these results, we analyzed the data of IL10 production following infection with influenza virus. Figure 2, panel B shows that the mean production of IL10, following PR8 infection, significantly differed between the male and female groups being almost 4 fold higher in males. These data suggest that a higher production of IL10 in males could be relevant in determining a decreased ability of males to control influenza virus infection [27]. They also suggest that the virus could interact with TLR8 to stimulate IL10 production. Similar to IFN-α response following TLR7 stimulation, the production of IL10 in response to TLR3, 7, 8 ligands or following infection with PR8 was not dependent on “in vivo” stimulation by sex hormones and/or Cortisol. We did not find any significant correlation between IL10 production and the plasma levels of sex hormones in male or female groups (data not shown), thus further confirming that metabolic pathways activated by the recognition of viral RNA are not under the influence of sex hormones.

### IL10 Production in Response to TLR9 Ligands and to HSV-1 Infection

**Figure 3:**
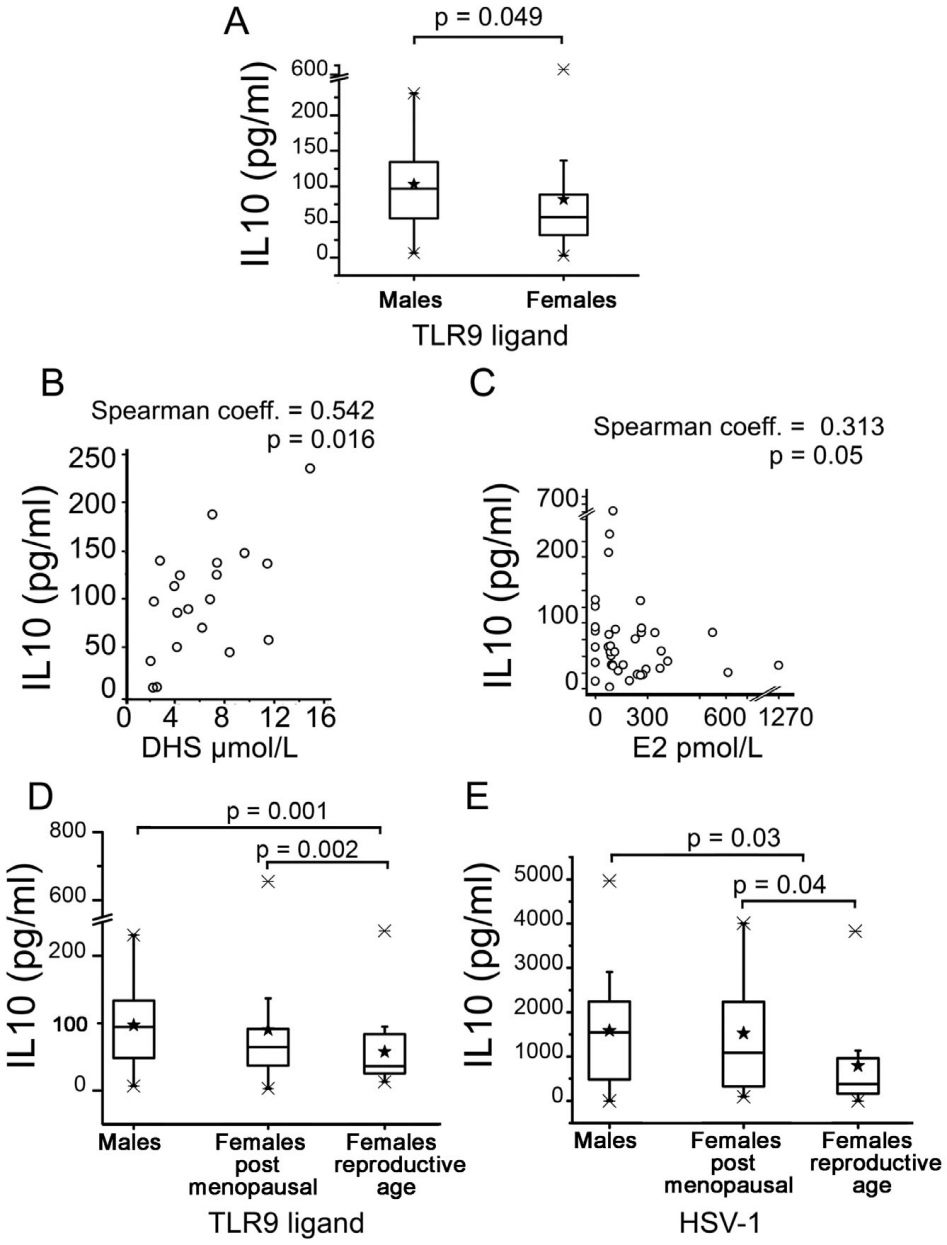
IL10 production by PBMCs following TLR9 stimulation or infection with HSV-1 and sex hormones-correlation. Panel A: IL10 production by PBMCs from males (n = 20) and females (n = 40) following stimulation with ODN 2006 as TLR9 ligand. Panel B: Spearman rank correlation analysis between IL10 production and DHS levels in the male group. Panel C: Spearman rank correlation analysis between IL10 production and E2 levels in the female group. Panel D: IL10 production by PBMCs from males (n = 20, mean age, years 47±14, range 22–62, females in post menopausal age (n = 20, mean age 59±7, range 48–71) and females in reproductive age (n = 20, mean age 33±9 range 25–54), following stimulation with CpG-ODN 2006 as TLR9 ligand. Panel E: IL10 production by PBMCs from males, females in post-menopausal age (n = 20, mean age 59±7, range 48–71), and females in reproductive age (n = 20, mean age 33±9, range 25–54) following infection with HSV-1. Data of IL10 production are calculated as mean IL10 production in stimulated cultures – mean IL10 production of unstimulated cultures. Data are presented as box-and-whisker plots, with boxes extend from the 25^th^ percentile to the 75^th^ percentile, with a horizontal line at the median while whiskers extend to the lowest and highest data point. Black stars indicate mean values. Statistical analysis was performed by two-tailed non-parametric Mann-Whitney test. Significance levels were fixed at p<0.05. Analysis of linear trends was performed using Spearman rank correlation. The level of statistical significance was set at p<0.05. IL10 concentrations in unstimulated or mock-infected cultures ranged between 2 and 68 pg/ml (mean 28.3±1.6). No significant differences between the groups were detected.

Synthetic oligonucleotides (ODN-2006) used as stimulant of TLR9 induced a 2.7±0.3 mean increase of IL10 production by PBMCs from all the subjects and again the production of IL10 was higher in males than females although the results did not reach high levels of significance (Figure 3 panel A). However, by using Spearman rank correlation we evidenced a linear trend between the production of IL10 in response to TLR9 stimulation and the plasma levels of sex hormones. Figure 3, panel B shows the strong correlation between IL10 production and plasma DHS levels in the male group (p = 0.01). In the female group the IL10 production in response to TLR9 stimulation was, in contrast, inversely correlated with E2 levels (Figure 3, panel C). These data suggest that the sensitivity to TLR9 ligands in vitro, at least in terms of IL10 production, could have been differentially modulated by sex hormones in males and females. Menopause in females is the result of a major reduction in female hormonal production by the ovaries. Based on the results of Spearman rank correlation analysis, we hypothesized that major differences in IL10 production could be detected as the result of comparison between the male group and a group of females in active reproductive age, with E2 levels ranging between 200–1500 nmol/L (Table S1). Thus, the female group was subdivided in females in reproductive age (n = 20, mean age 33±9, range 25–54) and females in post-menopausal age (n = 20, mean age 59±7, range 48–71) (Table S1) and the data of IL10 production were re-analyzed. Figure 3, panel D shows that significant differences in IL10 production came out from the comparison of males with females in reproductive age, whereas no statistically significant differences were evidenced between males and females in post-menopausal age thus suggesting that plasma levels of estrogens could have modulated the sensitivity to TLR9 ligands at least in terms of IL10 production. The possibility that the response to TLR9 could be influenced by age was also investigated. Spearman rank correlation analysis did not show any correlation between the IL10 production and age in the total group of subjects involved (Spearman K = 0.224, p = 0.08).

To check the biological relevance of these data, PBMCs from males and females were infected with HSV-1 and the production of IL10 was measured in culture supernatants after 48 hours from infection. When we compared the IL10 production between the male group and the whole group of females (including post-menopausal women), we observed results similar to those obtained following TLR9 stimulation: the production of IL10 was higher in the male group compared to females, but the results did not reach the significance (males = 1584±280; females = 1160±190). However the comparison of IL10 production in the male group with that of females in reproductive age revealed marked and significant differences between the groups with males being higher producer of IL10 (Figure 3, panel E).

These data reinforce the hypothesis that sex hormones modulate TLR9 response to DNA viruses and suggest that females in reproductive age could take advantage of the lower production of IL10 to clear the pathogen. However, in post-menopausal age when E2 levels are low or virtually absent and the production of IL10 following viral DNA trigger of TLR9 is not more modulated, the immune response of females is not different from that of males.

On the whole our data suggest that major differences in TLR sensitivity between the sexes do not concern the production of the factors involved in the differentiation of TH effector cells but rather they concern factors able to modulate either the differentiation as well as the inflammation program.

## Discussion

To investigate the molecular mechanisms underlying sex-related differences in viral disease susceptibility [12]–[17], we compared cytokine production by PBMCs from males and females following stimulation of TLRs more involved in viral recognition or following infection with two viruses largely spread among adult population, representative of RNA viruses (influenza virus) and DNA viruses (HSV-1). The choice of these viruses was justified by data from literature reporting an higher incidence and severity in males of seasonal or pandemic influenza virus infection [28]–[33]; in murine models of HSV-1 keratitis, mortality and virus reactivation were found more common in males [34] and, in humans, the duration of HSV-1-induced genital lesions was reported to be significantly associated with male sex [35].

We found evidences that the activation of TLR7 with Imiquimod induces a higher production of IFN-α in the female group compared to males. Sex-related differences in IFN-α production following TLR7 stimulation were reported also by Berghofer *et al*. [22] who ruled out any influence of estrogen signaling on TLR7 sensitivity: accordingly we did not show any correlation with plasma levels of sex hormones in the two groups. Although influenza virus induces IFN-α production through TLR7 activation the production of this cytokine following infection of PBMCs with the PR8 strain was comparable in males and females. In fact, as far other RNA viruses, influenza virus induces IFN-α production through different pathways including TLR8, TLR3 but also PRRs different from TLR [23], [36]–[38] which are likely not differently modulated in the two sexes. Accordingly no differences were found between males and females in the production of IFN-α following stimulation of TLR8 and TLR3.

TLR3 and TLR9 activation were shown to be crucial in protective immunity against HSV-1 being involved in viral recognition and IFN-α production [39]. In particular, molecular pathways activated by TLR3 were involved in protection against lethal HSV-1 infections in the central nervous system [25], [26]. As following TLR3 stimulation, we couldn’t find statistically significant differences in IFN-α production between males and females following TLR9 stimulation or infection with HSV-1.

Although IFN-α production has been involved in the different clinical course of HIV infection in females [40], our data suggest that this mechanism could not represent the single one able to justify the sex-related differences in viral infections.

Furthermore, with the only exception of increased TNF-α production by males following TLR3 stimulation, no other sex-related differences were found in the production of cytokines involved in inflammatory processes or in T cell differentiation and function either following TLR stimulation or virus infection. Indeed, the most striking difference between the sexes concerns the production of IL10, recognized as a major anti-inflammatory cytokine and as a negative regulator of innate and adaptive immune response [7].

IL10 was also reported to play a role in immune response to influenza and herpes viruses. In murine models of influenza virus infection it was shown to impair the initial T-helper cell functions required for effective antibody production against virus thus contributing to susceptibility to primary infection [27]. Infected female mice generate higher proinflammatory responses to influenza virus experiencing greater morbidity and mortality than males [41]; however they mount higher immune responses and are better protected against a subsequent heterosubtypic lethal virus challenge than males [42]. In humans higher plasma levels of IL10 were reported as an important marker correlated with the severity of the disease during the 2009 pandemic H1N1 [43].

How sex-related IL10 production influences the outcome of infection and/or the amplitude of humoral immune response in the two sexes has not been investigated. Our data show that following infection with influenza virus, PBMCs from males produce more IL10 than females. The stimulation of TLR8 induces higher production of IL10 in males compared to females. In contrast, IL10 production following TLR7 stimulation is comparable in the two sexes. Although TLR7 and TLR8 are often seen as closely related TLRs because of their phylogenetic relationship, it has been shown that the engagement of these receptors brings into play different signaling pathways in the maturation process of dendritic cells [44]. TLR8 response in terms of IL10 production is not correlated with plasma levels of sex hormones, but it could be related to a higher number of TLR8-expressing monocytes in males [45]. The mechanisms underlying the different sensitivity of TLR8 in males and females are currently under investigation.

The immune response to HSV-1 is also modulated by IL10 [46], [47]. Our data show that females, particularly in reproductive age, produce less IL10 than males, either following HSV-1 infection or stimulation of TLR9, the most important PRR recognizing HSV-1 DNA [24]. In contrast to TLR7 and TLR8, the IL10 production following TLR9 activation was negatively correlated with the levels of E2 in females and positively correlated with DHS levels in males. The mechanisms through which E2 modulates the response to TLR9 ligands are not known yet. Estrogens have been reported to induce the down regulation of TLR9 expression in Estrogen Receptor (ER)+ breast cancer cell lines [48], but nothing has been reported regarding the expression and function of TLR9 on myeloid and lymphoid cells. In contrast it is known that Testosterone increases the production of IL10 by CD4+ cells even though the involvement of TLR9 was not reported [49]. Based on our data we suggest that a higher production of IL10 following virus infection could be partially responsible for the worse outcome in males reported in murine models of HSV-1 infection [34] or could be involved in the longer duration of viral lesions in human males [35], [50]. Studies in large cohorts of HSV-1 infected individuals are needed to validate these hypotheses.

Finally we must observe that although the lower IL10-mediated inhibitory pathway in females following TLR8 and TLR9 stimulation can help to rapidly clear the infectious agent, a prolonged inflammation can be detrimental because it may cause host toxicity and tissue damage. Thus females are likely to be prone to worse outcomes of the disease in those viral infections whose pathogenesis is mainly related to immune-inflammatory reactions. Furthermore, since an excessive production of inflammatory cytokines and chemokines via TLR pathways is often associated with the outcome of autoimmune diseases [19], it is possible to speculate that sex-related differences in viral-induced TLR responses could play a role in determining the higher prevalence of autoimmune diseases in females.

## Materials and Methods

### Subjects

60 healthy blood donors, 40 females (mean age, years 42±13, range, years 25–71) 20 males (mean age, years 47±14, range 22–62) were enrolled in the study after they gave written informed consent. Table S1 summarizes the features of each group. The study has been reviewed and approved by the Institutional Ethical Committee of the Azienda Ospedaliero-Universitaria Careggi, Firenze (protocol n° 2010/0033781, ref. n. 88/10 AOUC Firenze).

### Cell Cultures

Peripheral blood mononuclear cells (PBMCs) were isolated from heparin-blood samples by using Ficoll-Paque density gradient (GE Healthcare), according to the manufacturer's recommendations and cultured in duplicate at 10^6^/cells/ml in 48-well flat-bottom plates in RPMI 1640 medium supplemented with L-glutamine, 10% heat-inactivated fetal bovine serum (FBS) (Celbio), in the presence or absence of: 10 µg/ml Poly (I:C) as TLR3 stimuli, 1 µg/ml Imiquimod as TLR7 stimuli, 1 µg/ml ssRNA40 as TLR8 stimuli, 5 µM CpG-ODN 2006 as TLR9 stimuli. All the TLR ligands were from InvivoGen. Culture supernatants were collected after 48 hours and stored at −20°C.

Human Embryonic Kidney (HEK)-293 cells, highly permissive to influenza virus infection [51], HEK-293 transfected with human (hu) TLR7, with huTLR8 gene and MOCK-transfected HEK293 cells were obtained from INVIVOGEN and cultured in DMEM 4.5 g/L glucose, antibiotics (penicillin/streptomycin), L-glutamine, 10% heat-inactivated FBS.

### Virus Production and Infection

Influenza A/Puerto Rico/8/34 H1N1 virus (PR8) and Herpes Simplex virus type I (HSV-1) were grown as follows: a) PR8 was grown in the allantoic cavities of 10-day-old embryonated chicken eggs. After 48 hours at 37°C, the allantoic fluid was harvested and centrifuged at 5000 rpm for 30 min to remove cellular debris; b) monolayers of VERO cells in 75-cm^2^ tissue culture flasks were infected with HSV-1 strain F at a multiplicity of infection (m.o.i.) of 0.01. After 48 hours at 37°C, infected cells were harvested with 3 freeze-and-thaw cycles, and cellular debris were removed with low-speed centrifugation. Titration of PR8 and HSV-1 was determined by standard plaque assay [52], [53].

For viral infection, PBMCs were incubated at 37°C in RPMI containing 10% FBS for 4 hours. Cells were then centrifuged for 10 min at 1800 rpm and suspended in 200 µl of RPMI. Each sample was challenged with PR8 or HSV-1 for 3 hours at 37°C, washed with PBS, and then incubated with medium supplemented with 2% FBS. Mock infection was performed at the same conditions for viral infection. Supernatant of infected cells was recovered and used for cytokines measurement and for viral titration.

A time-dependent response of TNF-α and IL10 production following viral infection showed 24 hours and 48 hours to be an optimal time of cytokine induction for influenza virus (3 m.o.i.) and HSV-1 (3 m.o.i.) infection, respectively. In these preliminary experiments, influenza virus titer measured at 24 hours p.i. was 4×10^5^ plaque-forming units (pfu)/ml, whereas HSV-1 virus titer measured at 48 hours p.i. was 3.6×10^4^ pfu/ml.

To verify that PBMCs from healthy donors were actually infected with PR8 or HSV-1 a PCR analysis was performed in the supernatant of infected cells at 24 or 48 hours p.i., respectively. Briefly, viral DNA (HSV-1) and viral RNA (H1N1) were extracted with the QIAamp DNA or QIAamp RNA mini kit according to the manufacturer’s instruction (QIAGEN, Milan, Italy). Reverse-transcription and amplification was carried out using the OneStep RT-PCR Kit according to the manufacturer’s instructions (QIAGEN). Viruses were detected by qualitative PCR. The primer sequences were as follows:

H1N1 F 5′AAGACAAGACCAATACTGTCACCTCT3’; R 5′ TCTACGATGCAGTCCACGCT3’ HSV-1 F 5′AGCGTCTTGTCATTGGCGAA3’; R 5′ TTTTCTGCTCCAGGCGGACT 3′.

The PCR products were analyzed on a 2% agarose gel stained with ethidium bromide and visualized under ultraviolet light. All the necessary precautions were taken to avoid contamination.

### Cytokine Measurement

Human IFN-α, TNF-α, IL10, IL12p40, IL12p70 were measured by Multiplex array using a BioPlex apparatus according to manufacturer’s instructions.

### TLR 7/8 Assay

HEK-293 transfected with human (hu) TLR7, with huTLR8 gene and MOCK-transfected HEK293 cells were stimulated with 0.5–5 µg/ml of Imiquimod, ssRNA40 or infected with PR8 (3 m.o.i.) as above reported. Supernatants were collected after 24 hours and IL8 levels were measured by Immunoplex array. Data from these experiments (Figure S2) show that Imiquimod interacts only with huTLR7 while ssRNA40 interacts with huTLR8. Viral infection induced IL8 production in either TLR-transfected and MOCK-transfected cells.

### Hormone Measurement

Estradiol (E2), Testosterone (TES), Dehydroepiandrosterone sulfate (DHS), Cortisol (COR) levels were measured in plasma samples by using the Immulite 2000 (Diagnostic Products), according to manufacturer’s instructions. Table S1 reports the median of plasma levels of sex hormones in all the groups.

### Statistical Analysis

Statistical analysis of data was performed by two-tailed non-parametric Mann-Whitney’s U test using SPSS 15.0 software. A two-tailed p<0.05 was considered statistically significant. Analysis of linear trends was performed using Spearman rank correlation. The level of statistical significance was set at p<0.05.

## Supporting Information

Figure S1 **TNF-α production by PBMCs from males and females following stimulation with TLR3 ligand.** PBMCs from males (n = 20) or from females (n = 40) were stimulated with Poly (I:C) as TLR3 ligand, TNF-α was measured in culture supernatants by immunoplex array. Data for each donor are calculated as mean TNF-α production in stimulated cultures – mean IFN-α production of unstimulated cultures. Data are presented as box-and-whisker plots, with boxes extend from the 25^th^ percentile to the 75^th^ percentile, with a horizontal line at the median while whiskers extend to the lowest and highest data point. Black stars indicate mean values. Statistical analysis was performed by two-tailed non parametric Mann-Whitney test. Significance levels were fixed at p<0.05.
(TIF)

Figure S2 **IL8 production induced by Imiquimod, ssRNA40 and PR8 influenza virus on HEK-293 cells.** 10^5^ TLR7-HEK-293, TLR8-HEK-293 or MOCK-transfected HEK-293 cells were stimulated with 0.5–5 µg/ml of Imiquimod, ssRNA40 or infected with PR8 (3 m.o.i.). Supernatants were collected after 24 hours and IL8, IFN-α levels were measured by Immunoplex array. The Figure shows IL8 production induced by optimal concentrations (1 µg/ml) of Imiquimod or ssRNA40. Data from 3 different experiments (mean ± SE) are shown.
(TIF)

Table S1 **Male and female groups enrolled in the study.** 60 healthy blood donors, 40 females and 20 males were enrolled in the study after they gave written informed consent. Cortisol (COR), Estradiol (E2), Testosterone (TES), Dehydroepiandrosterone sulfate (DHS) levels were measured in plasma samples by Immulite 2000. Reference values are: COR = 138−690 nmol/L; DHS = 2.17−15.2 µmol/L; TES = 6−55 nmol/L (depending on age); E2 = 0−1468 pmol/L (depending on menstrual cycle).
(XLS)
