# Supplementary figures and images for: Sex Differences in the Response to Viral Infections: TLR8 and TLR9 Ligand Stimulation Induce Higher IL10 Production in Males
Source: PLoS One. 2012 Jun 29;7(6):e39853. doi: 10.1371/journal.pone.0039853 (PMC3387221; doi:10.1371/journal.pone.0039853)

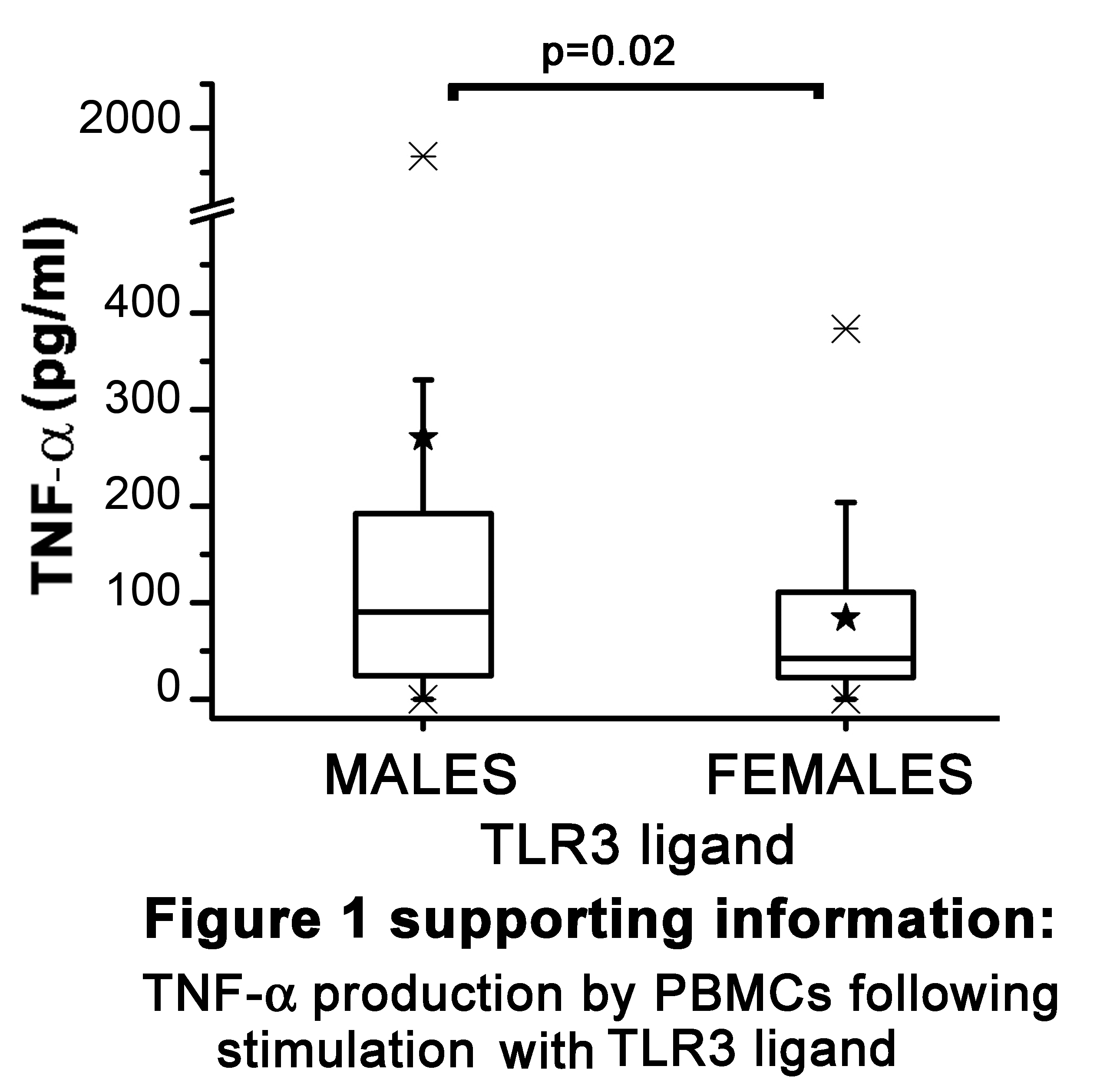

Supplement: Figure S1 — TNF-α production by PBMCs from males and females following stimulation with TLR3 ligand. PBMCs from males (n = 20) or from females (n = 40) were stimulated with Poly (I:C) as TLR3 ligand, TNF-α was measured in culture supernatants by immunoplex array. Data for each donor are calculated as mean TNF-α production in stimulated cultures – mean IFN-α production of unstimulated cultures. Data are presented as box-and-whisker plots, with boxes extend from the 25th percentile to the 75th percentile, with a horizontal line at the median while whiskers extend to the lowest and highest data point. Black stars indicate mean values. Statistical analysis was performed by two-tailed non parametric Mann-Whitney test. Significance levels were fixed at p<0.05. (TIF) [file pone.0039853.s001.tif]

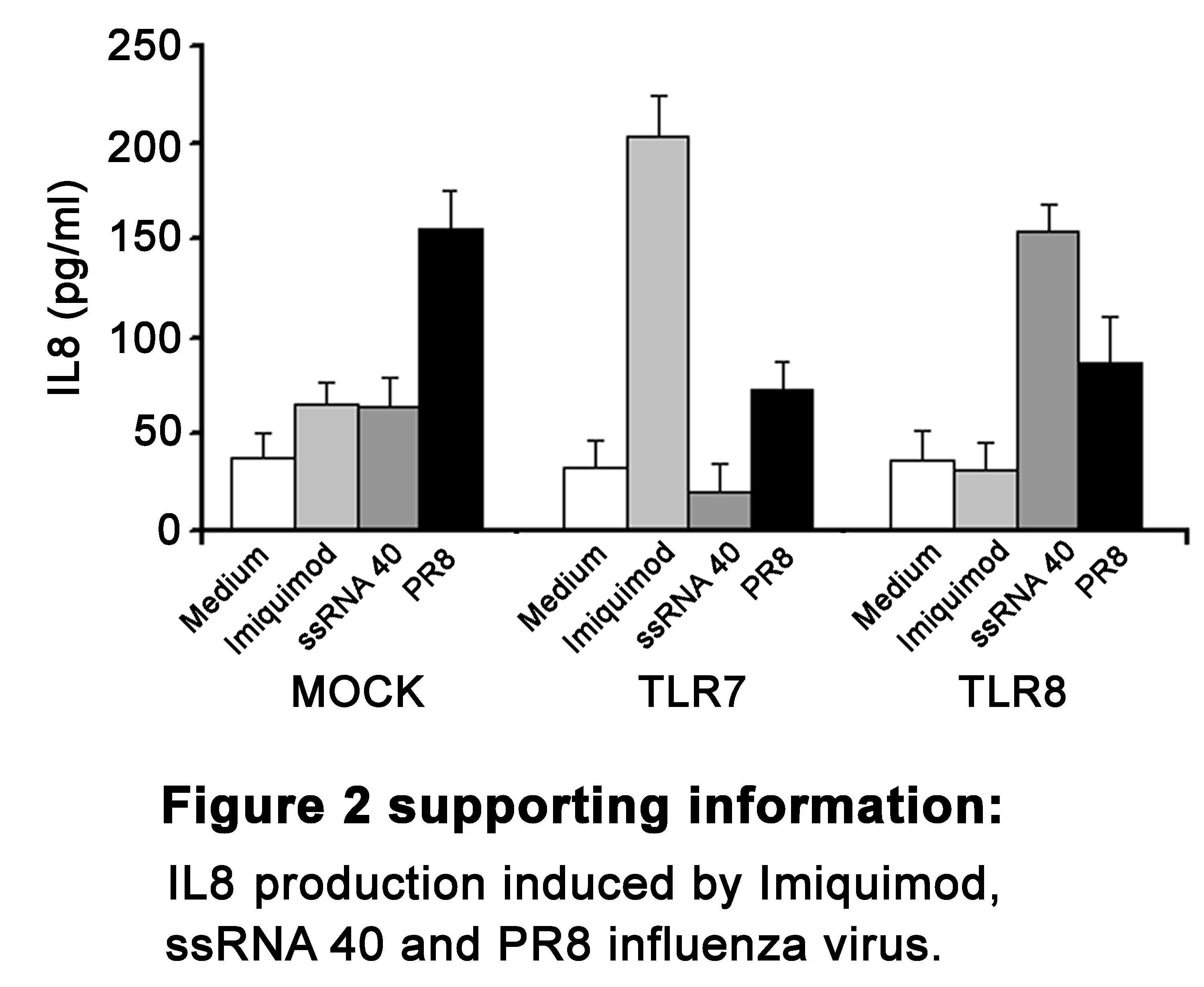

Supplement: Figure S2 — IL8 production induced by Imiquimod, ssRNA40 and PR8 influenza virus on HEK-293 cells. 105 TLR7-HEK-293, TLR8-HEK-293 or MOCK-transfected HEK-293 cells were stimulated with 0.5–5 µg/ml of Imiquimod, ssRNA40 or infected with PR8 (3 m.o.i.). Supernatants were collected after 24 hours and IL8, IFN-α levels were measured by Immunoplex array. The Figure shows IL8 production induced by optimal concentrations (1 µg/ml) of Imiquimod or ssRNA40. Data from 3 different experiments (mean ± SE) are shown. (TIF) [file pone.0039853.s002.tif]
